# Sodium-Glucose Co-Transporter 2 Inhibitors May Change the Development of Urinary Tract and Hematological Malignancies as Compared With Dipeptidyl Peptidase-4 Inhibitors: Data of the Post-Hoc Analysis of a Nationwide Study

**DOI:** 10.3389/fonc.2021.725465

**Published:** 2021-10-28

**Authors:** György Rokszin, Zoltán Kiss, Gábor Sütő, Péter Kempler, György Jermendy, Ibolya Fábián, Zoltán Szekanecz, Gyula Poór, István Wittmann, Gergő Attila Molnár

**Affiliations:** ^1^ RxTarget Ltd, Szolnok, Hungary; ^2^ Second Department of Medicine and Nephrology-Diabetes Centre, University of Pécs Medical School, Pécs, Hungary; ^3^ Department of Internal Medicine and Oncology, Faculty of Medicine, Semmelweis University, Budapest, Hungary; ^4^ Bajcsy-Zsilinszky Hospital, Budapest, Hungary; ^5^ Faculty of Mathematics, University of Veterinary Medicine, Budapest, Hungary; ^6^ Department of Rheumatology, Faculty of Medicine, University of Debrecen, Debrecen, Hungary; ^7^ National Institute of Rheumatology and Physiotherapy, Budapest, Hungary

**Keywords:** diabetes mellitus type 2, cancer, antidiabetic treatment, dipeptidyl peptidase (DPP)-4 inhibitors, sodium-glucose co-transporter 2 (SGLT 2) inhibitors

## Abstract

**Background:**

In diabetes mellitus, during the last years, cancer became of equivalent importance as a cardiovascular disease in terms of mortality. In an earlier study, we have analyzed data of the National Health Insurance Fund (NHIF) of Hungary with regards all patients treated with sodium-glucose co-transporter 2 (SGLT2) inhibitors (SGLT2is) *vs*. those treated with dipeptidyl peptidase-4 (DPP-4) inhibitors (DPP-4is) in a given timeframe. In propensity score-matched groups of SGLT2i- *vs*. DPP-4i-treated patients, we found a lower incidence of cancer in general. In this post-hoc analysis, we aimed to obtain data on the incidence of site-specific cancer.

**Patients and Methods:**

All patients starting an SGLT2i or a DPP-4i between 2014 and 2017 in Hungary were included; the two groups (SGLT2i *vs*. DPP-4i) were matched for 54 clinical and demographical parameters. The follow-up period was 639 *vs*. 696 days, respectively. Patients with a letter “C” International Classification of Diseases, 10th Revision (ICD-10) code have been chosen, and those with a known malignancy within a year before the onset of the study have been excluded from the analysis.

**Results:**

We found a lower risk of urinary tract [HR 0.50 (95% CI: 0.32–0.79) p = 0.0027] and hematological malignancies [HR 0.50 (95% CI: 0.28–0.88) p = 0.0174] in patients treated with SGLT2i *vs*. those on DPP-4i. Risk of other types of cancer (including lung and larynx, lower gastrointestinal (GI) tract, rectum, pancreas, non-melanoma skin cancers, breast, or prostate) did not differ significantly between the two groups. When plotting absolute risk difference against follow-up time, an early divergence of curves was found in case of prostate, urinary tract, and hematological malignancies, whereas late divergence can be seen in case of cancers of the lung and larynx, the lower GI tract, and the breast.

**Conclusions:**

Urinary tract and hematological malignancies were less frequent in patients treated with SGLT2i *vs*. DPP-4i. An early *vs*. late divergence could be observed for different cancer types, which deserves further studies.

## Introduction

Data of the last decades indicated that cardiovascular (CV) mortality is the leading cause of death in type 2 diabetes mellitus (T2DM). This has become a paradigm of diabetology and even changed the guidelines (1–3). However, a recent paper drew the interest to non-CV causes of death. It has shown that the leading causes of mortality have changed substantially in England between 2001 and 2018. While mortality attributable to CV events has dramatically changed, there was a large increase in mortality due to dementia, and the rate of cancer mortality did not change markedly. In 2001, CV death was responsible for 45.4% and 45.2% of the overall mortality in men and women, respectively; by 2018, this proportion decreased to 27.0% and 24.0%, respectively. On the other hand, in 2001, the relative contribution of cancer to overall mortality was 25.8% and 19.4% in males and females, respectively; and by 2018, this proportion rose to 33.4% and 27.5%. Consequently, these data confirm that cancer has become the leading cause of mortality in this population (4).

Beyond glycemic parameters and lifestyle, antidiabetic medications may have an impact on cancer risk as well (5). Since overfeeding and obesity contribute to an excess risk with regard to malignancies, it seems reasonable that agents leading to weight loss could lead to a decrease in the risk of cancer. In fact, weight loss and starvation are proposed in the literature to be used as an anticancer strategy (6).

Therefore, comparison of the effect of sodium-glucose co-transporter 2 (SGLT2) inhibitors (SGLT2is) as a medication associated with weight loss and metabolic off-load with dipeptidyl peptidase-4 (DPP-4) inhibitors (DPP-4i) as weight-neutral agents could be of interest also in terms of cancer.

In a previous publication, our workgroup compared patients treated with SGLT2i with patients treated with DPP-4i in a nationwide analysis (7). After a thorough propensity match for 54 clinical parameters, we obtained more than 18,000 cases in each group. We compared the risk of CV events, amputation, hospitalization for heart failure, death, the risk of cancer. We found a significantly lower risk of cancer in general for patients on SGLT2i *vs*. those on DPP-4i. In a further analysis, we conducted a cancer site-specific analysis using these cohorts. The results of this analysis are demonstrated in the present paper.

## Materials and Methods

### Research Design

We obtained data from the National Health Insurance Fund (NHIF) of Hungary in an anonymized manner. All patients with T2DM who initiated an SGLT2i or a DPP-4i between August 1, 2014, and July 1, 2017, were included in the study; thus, a nationwide coverage could be achieved. Subsequently, a propensity score matching for 54 demographical and clinical parameters (including sex, age at diagnosis, age at the index date, comorbidities, and antidiabetic and CV medications) has been performed in a 1:1 ratio. This resulted in two well-matched groups. A detailed description of the study was previously published in an earlier paper (7). The study period covered the time between August 2014 and July 2017, providing an average follow-up of 639 and 696 days for the SGLT2i and DPP-4i arms, respectively. Patients with any malignant disease within 1 year prior to the index date were excluded from the analysis of cancer (7). One major difference between the present publication and our previous publication is that the earlier paper also included a comparison of a group with SGLT2i as an add-on treatment to DPP-4i *vs*. another group, where DPP-4i was substituted for an SGLT2i. This SGLT2i add-on *vs*. switch comparison could not be performed here in terms of cancer events because of the low number of events. Thus, the present paper only provides comparison of SGLT2i clear- *vs*. DPP-4i clear-treated groups.

In the present analysis, the International Classification of Diseases, 10th Revision (ICD-10) codes between C00 and C99 have been used. The first letter “C” ICD code and the corresponding date were recorded. Afterwards, we were looking for a subsequent letter “C” ICD code until June 2019 (end of study) either in outpatient or in inpatient reports, irrespective of the time interval between the two ICD codes. In case the patient had more than one letter “C” ICD code, the code of a primary cancer with the highest prevalence in the records was the main cancer diagnosis. The first mention of the “C” ICD code in inpatient records was regarded as the time of diagnosis of the malignancy. Secondary (metastatic) cancer was taken as a main diagnosis only in case the patient had a C77-80 ICD code, without having a primary cancer code registered in any of the records. If a patient had even ICD codes for two cancer types, their latest report was chosen as the main cancer diagnosis.

Cancer sites were grouped into larger categories on an anatomical basis to provide a sufficient amount of data in the individual groups; hence, data were obtained for the head and neck, the lung and larynx, the upper gastrointestinal (GI) tract, the lower GI tract, the rectum, the hepatobiliary system, the pancreas, melanoma, non-melanoma skin cancers, cancers related to the female sex organs, the breast, male sex organs, the prostate, the urinary tract, hematological cancers, and other unspecified cancer sites as well as secondary tumors (metastases). For reasons of data security, cancer sites with less than 10 cases cannot be presented in the current paper to preserve anonymity of the patients.

### Statistical Methodology

For the propensity score matching, a caliper of 0.2 has been used. We performed survival analysis comparing the propensity score-matched SGLT2i *vs*. DPP-4i groups. A Cox proportional hazards model with time since index date was used to obtain hazard ratios, the 95% confidence intervals (CIs), and p-values. Selected endpoints were also graphed using the Kaplan–Meier-type survival curves. Also, the absolute risk difference between the SGLT2i arm and the DPP-4i arm has been calculated and graphed as a function of follow-up time (8). A negative difference refers to a lower risk in the SGLT2i arm. The statistical software R (version 3.6.1) was used for the statistical analyses.

## Results

After the propensity score matching, two groups of 18,583 patients in each arm have been obtained. The groups had an approx. 48% of female patients, their age at diagnosis of diabetes, the age at index date, the duration of diabetes, and previous comorbidities, antidiabetic and CV medications were properly matched in both groups (**Table 1** and (7)].

**Table 1:** Selected clinical parameters of the SGLT2i arm *vs*. the DPP-4i arm after the propensity score matching.

Parameter	SGLT2i arm	DPP-4i arm	SMD
Number of cases	18,583	18,583	
Female gender, n (%)	8,900 (47.9)	8,934 (48.1)	0.004
Age at diagnosis of DM (mean (SD))	54.4 (9.5)	54.7 (10.8)	0.030
Age at index date (mean (SD))	59.6 (10.0)	59.8 (11.6)	0.019
Days from diagnosis to index (mean (SD))	1,913 (958)	1,875 (980)	0.039
Cancer in the past medical history, n (%)	1.297 (7.0)	1.305 (7.0)	0.002

Data are taken from reference 7, an Open Access paper, upon written permission (7).

DM, diabetes mellitus; n, number of cases; SMD, standardized mean difference; SGLT2i, sodium-glucose co-transporter 2 inhibitor; DPP-4i, dipeptidyl peptidase-4 inhibitor.

**Figure 1:**
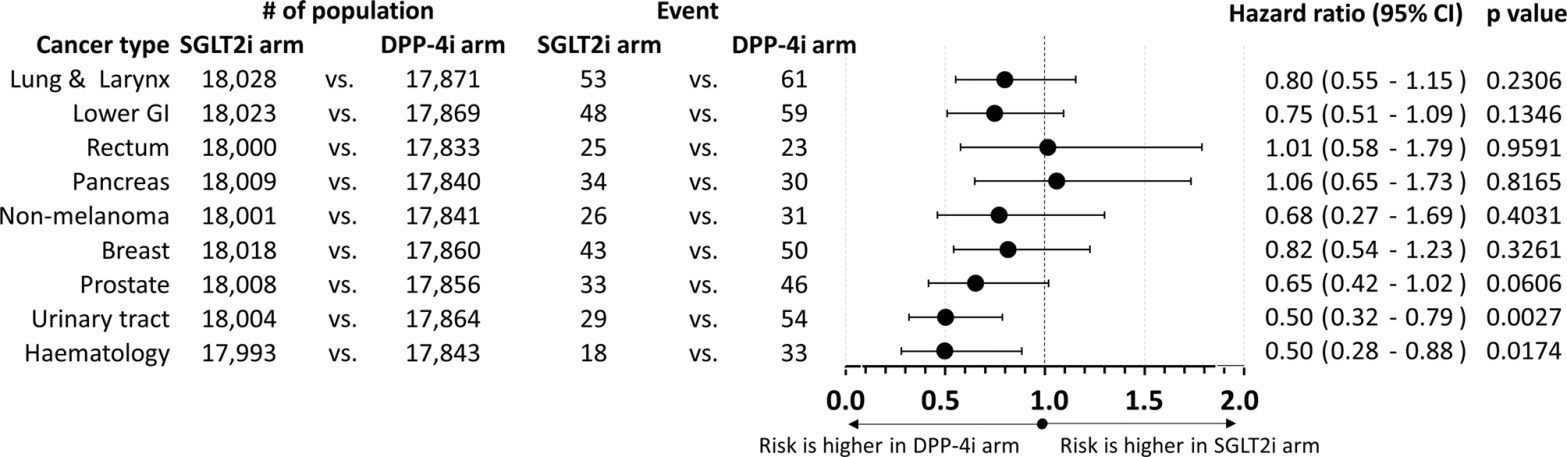
Forest plot on the comparison of SGLT2i and DPP-4i arms and the risk of individual cancer types. Hazard ratios as well as their 95% confidence intervals are shown; p-values are also reported. Hazard ratios below 1.0 indicate that the risk is higher in the DPP-4i arm, whereas hazard ratios above 1.0 signify that the risk is higher in the SGLT2i arm. GI, gastrointestinal tract; SGLT2i, sodium-glucose co-transporter 2 inhibitor; DPP-4i, dipeptidyl peptidase-4 inhibitor.

We have compared the number of cancer events in patients in the SGLT2i arm to patients in the DPP-4i arm. Different locations were studied. The hazard ratios and the 95% CIs of several locations are depicted in **Figure 1**. Because of data security reasons and to provide anonymity of the patients, groups with less than 10 events in any arm (including cancers of the head and neck region, the upper GI tract, the hepatobiliary system, melanoma, the male sex organs, and metastatic cancer cases) had to be excluded from the analysis; thus, they are not shown in **Figure 1**. Hence, the overall number of events in the SGLT2i *vs*. DPP-4 arm (334 *vs*. 450) has to be lower than the number of total cancer events reported in our previous paper (408 *vs*. 506 events) (7).

When comparing SGLT2i *vs*. DPP-4i patient groups, there was no significant difference in the risk of cancers affecting the lung and larynx, the lower GI tract, the rectum, the pancreas, the non-melanoma skin cancers, the breast or the prostate, the female sex organs, and other cancers. The HR values were numerically less than 1.0 for most cancer types, except for the rectum (1.01), the pancreas (1.06, as seen in **Figure 1**), or hepatobiliary (1.17, not shown in **Figure 1**) cancer. Again, we must declare that there was no significant difference concerning these values, either.

On the other hand, we found a significantly lower risk of urinary tract cancers [HR 0.50 (95% CI: 0.32–0.79)] and of hematological malignancies [HR 0.50 (95% CI: 0.28–0.88)] in the case of patients treated with SGLT2i *vs*. those treated with DPP-4i. We found no significant increase of risk for any cancer sites in the group treated with SGLT2i *vs*. those treated with DPP-4i.

**Figure 2:**
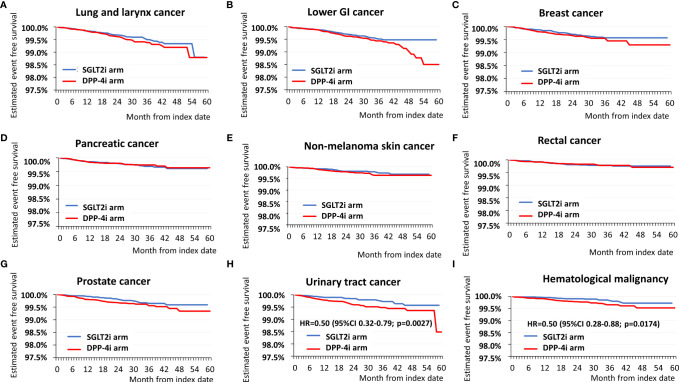
Kaplan-Meier survival curves of selected cancer sites: **(A)** lung and larynx cancer, **(B)** lower GI tract cancer, **(C)** breast cancer, **(D)** pancreatic cancer, **(E)** non-melanoma skin cancer, **(F)** rectal cancer, **(G)** Prostate cancer, **(H)** urinary tract cancer, **(I)** hematological malignancies. The blue curves show cancer-free survival in the SGLT2i arm, the red curves the cancer-free survival in the DPP-4i arm. In case of significant difference in hazard, also the exact hazard rates, 95% confidence intervals and p values are presented. It must be noted that the axis of survival is on the same scale for each graph, enabling visual comparisons of curves of different cancer sites.

Subsequently, we analyzed survival data using the Kaplan–Meier survival curves and Cox regression analysis. Selected curves are depicted in **Figure 2**. **Figures 2A–C** show data concerning lung and laryngeal cancer, lower GI tract cancer, and breast cancer, respectively. **Figures 2D–F** depict pancreatic, non-melanoma skin, and rectal cancers, respectively; while **Figures 2G–I** show prostate, urinary tract, and hematological malignancies. It must be noted that data have been scaled to the same axis. Both urinary tract malignancies and hematological malignancies show an early divergence (of curves from approx. the sixth to 10th months) with a statistically significant difference. Furthermore, one may observe that curves on **Figures 2A–C, G** also seem to diverge, although there was no statistical difference regarding the two groups and risk of cancer at these cancer sites.

**Figure 3:**
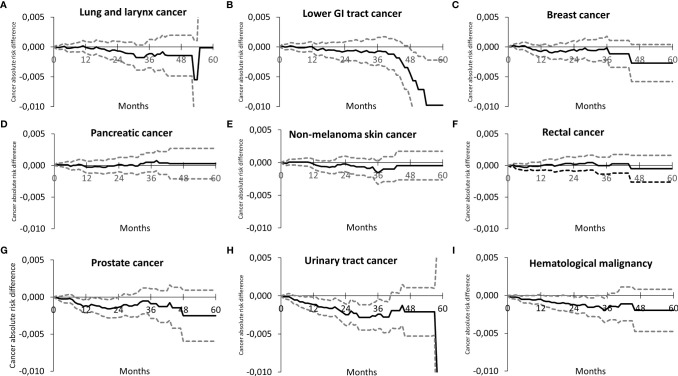
Absolute risk difference between the SGLT2i arm and the DPP-4i arm as a function of time for different cancer sites. It is to be noted that a negative difference indicates a lower actual risk for the SGLT2i arm as compared to the DPP-4i arm. The dotted lines represent the corresponding 95% confidence intervals. The order of individual sites is the same as in **Figure 2**, i.e. **(A)** lung and larynx cancer, **(B)** lower GI tract cancer, **(C)** breast cancer, **(D)** pancreatic cancer, **(E)** non-melanoma skin cancer, **(F)** rectal cancer, **(G)** Prostate cancer, **(H)** urinary tract cancer, **(I)** hematological malignancies.

To be able to visualize the difference in divergence of the survival curves, the absolute risk difference was plotted for cancer types depicted in **Figures 1**, **2** against follow-up time. **Figure 3** shows in the same structure as **Figure 2**, that the lung and larynx, lower GI tract, and breast cancers have a late risk reduction (**Figures 3A–C**); while in the case of prostate, urinary, and hematological malignancies, the risk already seems to decrease between 2 and 12 months (**Figures 3G–I**). **Figures 3D–F** are in line with the overlapping survival curves seen in **Figure 2**, for pancreatic, non-melanoma skin, and rectal cancers, respectively.

## Discussion

Our paper provides a nationwide analysis of cancer cases regarding SGLT2i *vs*. DPP-4i antidiabetic treatment. We found that in patients on SGLT2i, the hazard of development of urinary tract cancer or hematological malignancy was half that of the hazard in patients taking DPP-4i. There was no significant difference between the groups in other types of cancer, but—with exception of rectal, hepatobiliary, and pancreatic cancers—the point estimates for cancer were less than 1.0 in the SGLT2i arm for these cancer types.

T2DM itself is associated with an overall higher risk of cancer. This is attributable to multiple factors, such as hyperglycemia-induced free radicals and free-radical-associated mutagenesis, inhibition of apoptosis, altered insulin signaling, effects of overweight or obesity, poor diet, and lack of physical activity (9, 10), leading to higher risk of breast, endometrial, and colorectal cancers (9, 11, 12).

Beyond intrinsic properties of DM, antidiabetic medications, too, may have an influence on the risk of cancer. One should bear in mind that when comparing two treatments and observing a difference between them, the difference observed may be the consequence of the protective effect of one agent (in this case the SGLT2i), a potential harmful effect of the other agent (in this case the DPP-4i), or the combination of both. Therefore, it does make a large difference which classes of agents are compared.

DPP-4is have—among others—insulin secretion-inducing properties (13). Since insulin *per se* is a mitogenic factor, insulin secretagogues could theoretically induce higher cancer rates (14). Furthermore, as the DPP-4 enzyme is a putative enzyme that is able to cleave several peptides, it is feasible that it may interfere with several peptide-signaling pathways. As one of these pathways, DPP-4 inhibition may alter chemokine signaling (e.g., that of CXCL-12 and its downstream receptor CXCR4). This may in turn change the activity of the mammalian target of rapamycin (mTOR) pathway, which could theoretically result in tumor cell proliferation and metastasis formation (15).

Some *ex vivo* data indeed suggest that potential targets of DPP-4/CD26 could interfere with chemokine signaling, and the expression of DPP-4/CD26 seems to be associated with cancer aggressiveness and outcomes, although data may be controversial concerning whether it may promote or inhibit cancer growth (15). In a mouse model of breast cancer, inhibition of DPP-4 promoted metastasis formation, while metformin had an inhibitory effect on it by influencing mTOR-related signals (16).

The clinical data regarding DPP-4i and cancer risk or outcomes are controversial. A Medicare database search found improved survival of lung and colorectal cancer patients on DPP-4i. This effect was further improved by the concomitant use of metformin (17). Another database analysis found an increased survival in users of DPP-4i among patients with prostate cancer but not among patients with breast or pancreas cancer (18). A study on propensity score-matched groups of DPP-4i users (n = 769) *vs*. metformin users (n = 769) found no difference in cancer risk, although the overall cancer incidence was relatively low (20 *vs*. 33 cases) (19). In the J-Brand prospective real-world study, alogliptin use was associated with a higher risk of cancer, however, not in the multivariate Cox regression analysis, where age and smoking were associated with cancer (20). A meta-analysis of 72 studies found no increased risk of cancer for DPP-4i users [relative risk (RR): 1.01 (0.91–1.12)]. There was no difference between DPP-4i users and non-users regarding major cancer types (GI, respiratory, urinary, etc.). Concerning individual cancer types, the risk was only significantly different for rectal cancer with an RR of 0.41 (0.18–0.95), thus rather showing a protective effect, if any (21). In another meta-analysis, involving 157 trials, no increase in risk of cancer was found in DPP-4i users; indeed, as compared with those of placebo, the overall cancer risk [OR: 0.90 (0.82–0.99)] and colorectal cancer risk [OR: 0.70 (0.52–0.94)] was lower (22). In a meta-analysis of trials on advanced lung and colorectal cancers, the progression-free survival was higher in DPP-4i users than in the controls (23).

SGLT2i was studied on its effects on cancer under *in vitro* or *ex vivo* circumstances. Both SGLT1 and SGLT2 may help in providing excess energy to cancer cells; thus, their inhibition may result in the suppression of cancer growth (24). Both SGLT2 and SGLT1 are described to be present in the brain, prostate, and pancreatic cancers; SGLT1 are present on top in ovaries and head and neck cancers; while SGLT2 present on top also in lung cancer. This is verified not only at mRNA or protein levels but also using a α-methyl-4-deoxy-4-[^18^F]fluoro-d-glucopyranoside (Me4FDG) PET-CT scan. This may be important not only as a diagnostic tool, but SGLT2i may theoretically play an additional role as an adjuvant anticancer treatment. While DPP-4i is regarded as weight-neutral, SGLT2i generally leads to a clinically significant weight loss, contributing to possible anticancer properties (24, 25).

In a meta-analysis, there was no difference in the overall incidence of cancer between SGLT2i and comparators [OR: 0.98 (0.77–1.24)]. In a pre-specified subgroup analysis, SGLT2i was compared with DPP-4i, and the observed difference was not significant [OR: 0.67 (0.19–2.36)] (26). An important recent piece of evidence originates from a post-hoc analysis of the DAPA-CKD study. Their data suggest that similar to our observation on SGLT2i presented here, dapagliflozin was associated with a 58% lower cancer mortality [HR: 0.42 (0.19–0.97)] (27). The fact that this lower cancer mortality was not present in comparison with one particular class of antidiabetic agents but in comparison with placebo and standard-of-care treatment suggests that this effect is a unique feature of the SGLT2i dapagliflozin.

Cancers of the urinary tract were an important fear at the time of start of marketing of SGLT2i medications. It was feared that urine with a high glucose content would stay in the bladder for several hours, thus resulting in carcinogenesis *in loco*. On top of this, in documents filed with dapagliflozin for a Food and Drug Administration (FDA) new drug application, the number of bladder cancer cases was numerically higher for dapagliflozin (9/5,478 *vs*. 1/3,156) (28). A network meta-analysis showed no increase in the risk of bladder cancer or renal cancer (29). However, in the same paper, with a pairwise meta-analysis approach, a higher risk of bladder cancer was found [HR: 3.87 (1.48–10.08)] (29). In the CANVAS study, there was no difference between canagliflozin- and placebo-treated cases; however, in the EMPA-REG OUTCOMES trial, a numerically higher portion of the patients had bladder cancer (9 *vs*. 0); however, the overall incidence (9/7,028) was low (30).

In our study, the number of cases with urinary tract malignancies in general was not higher; in fact, and surprisingly, it was significantly lower in the SGLT2i *vs*. DPP-4i group. We should emphasize that this is true for a combined urinary tract cancer endpoint including bladder and renal carcinoma. We believe that the inhibition of uptake of glucose in cells lining the urinary tract could yield protection even against the high glucose-driven carcinogenesis. The discrepancy between our real-world data and data of randomized controlled trials (RCTs) may in part also be the consequence of detection bias and time-lag bias that can be problematic in RCT studies. A higher risk of genitourinary infections in SGLT2i-treated patients may also draw attention to other diseases of the urinary tract, thus resulting in detection bias in RCT (28). In our case, different types of bias were eliminated by the research design, the real-file setting, exclusion of patients with events within a year prior to the index date, and other statistical measures.

Probably the most striking finding in our study was the significantly lower risk of hematological malignancies in the SGLT2i arm as compared with the DPP-4i arm [HR: 0.50 (0.28–0.88)]. It has not yet been studied extensively whether SGLT2 is expressed in lymphoid or myeloid cells. According to one study using quantitative polymerase chain reaction (PCR; TaqMan), sodium myo-inositol co-transporter (SMIT), and SGLT5, and according to Figure 2B of that paper, to a minor extent, also SGLT2 is expressed in the tonsil as a part of the lymphatic system (31). According to data of The Human Protein Atlas, mRNA expression is present; however, no protein expression has been verified for total peripheral blood mononuclear cells, more specifically T lymphocytes and NK cells. Also, immune cells of the heart, T cells and macrophages of the kidney, T cells and B cells of the liver, macrophages of the lung, T cells and macrophages of the skin, and regulatory T cells, memory CD4 and CD8 T cells, and NK cells of flow cytometric analysis of blood are shown to exert weak SGLT2 RNA positivity according to The Human Protein Atlas (32, 33). On the other hand, SGLT1 does not seem to be expressed in blood cells (32). No data in the literature are available for SGLT2 expression in human hematological malignancies yet. As for DPP-4is, in a meta-analysis, there was no increased risk of hematological malignancies [RR: 1.05 (0.65–1.68)], leukemia [RR: 0.93 (0.45–1.93)], lymphoma [RR: 1.29 (0.67–2.49)], or Hodgkin’s disease [RR: 0.20 (0.01–4.13)] (21). In fact, data are available for DPP-4is vildagliptin and saxagliptin, but not for sitagliptin, alogliptin, or linagliptin to inhibit cell proliferation of myeloma cells, probably through modulation of DPP-8 (34). Both agents were available at the time of our analysis in Hungary. We believe that further research is warranted to explain differences in the risk of hematological diseases between the SGLT2i and DPP-4i arms.

Another interesting piece of evidence arising from our study concerns the shape of the Kaplan–Meier survival curves. First, we do acknowledge that results are only significant for urinary tract cancers and hematological malignancies. Apart from those, if only the form of separation of the curves is considered, it can be observed that in **Figures 2A–C**, the curves show a late separation, in the period of months 18–30; concerning **Figures 2D–F**, the curves overlap, and for **Figures 2G–I**, one may observe a relative early divergence of the curves (from approx. month 6). In a survival analysis, divergence of the curves (overlapping curves, crossing, diverging, and then converging curves and clearly separating curves) may be important (35, 36). Little is known, however, about the difference between early separating and late separating survival curves. In the field of oncology, late separation of the survival curves is suggested to be present in case of cancer immunotherapy; it is believed to be due to a benefit in patients with a slow disease progression (37). The difference in divergence of the curves may require multiple hypothetical explanations: i) cancers with early separation may have a faster proliferation and progression, while those with late separation may have a lower rate of cell proliferation; therefore, the beneficial effect of treatment only manifests later; ii) there are differences between tumor types, concerning the mode of their reaction to the given medication (in this case SGLT2i *vs*. DPP-4i); or iii) the medications are able to influence different pathological abnormalities in different cancer cell types. Since the evolution of cancers is now regarded as multifactorial, i.e., it involves mutagenic and mitogenic changes (38), early divergence of the curves may be related to an influence on mitogenic potency, and late diverging curves may arise as a consequence of an effect of mutagenesis.

One of the factors explaining at least a part of the potential beneficial impact of SGLT2i on cancer is their metabolic effects. Intermittent fasting or starvation may have a positive effect on the risk of cancer (6). Approaches targeting glucose metabolism may also alter anticancer activity of the immune system, by affecting the amount and activity of tumor-infiltrating CD8+ T lymphocytes and other subsets of white blood cells (39). Tumor cells may produce reactive oxygen species (ROS), and these activate hypoxia-inducible factor 1 alpha (HIF-1α), nuclear factor kappa-B (NFκB), and consequent lactate production in the adjacent fibroblasts, which in turn is associated with faster growth of the cancer cells (40). SGLT2i has been shown to lead to a lower activity of HIF-1α in the kidneys (41–43). Data regarding the kidneys suggest that hyperglycemia may lead to an abnormal metabolism with high hexokinase and pyruvate kinase M2 activity, low Sirtuin-3 levels, an activated STAT3, and HIF1α signaling and an aberrant glycolysis similar to the Warburg effect in cancer cells. Furthermore, empagliflozin but not insulin led to normalization of these signaling anomalies (44). Based on these facts, it seems feasible that SGLT2i can block HIF-1α and thereby change metabolism in cancer cells or stromal fibroblasts, and we might speculate that they could lead to a slower cell division and a lower rate of mitogenesis, which could contribute to the results seen in our study on an early divergence of the cancer-survival curves.

Summing up, in our nationwide study comparing patients treated with SGLT2i *vs*. DPP-4i, we found that in the background of lower overall cancer incidence with SGLT2i treatment, there is a significant lower risk for urinary tract cancers and hematological malignancies. For most other cancer sites, the tendency favors SGLT2i treatment; however, results between the groups are not statistically significant. One can also observe a difference in the early *vs*. late divergence of the survival curves between different organs.

## Limitations

Despite being a nationwide study, due to the selected nature of population and the given time range, the number of cases that could be included into the study was limited. The sample size was further decreased by the propensity score matching approach. Thus, the number of cancer events was low, and this might have accounted in part for the wide CI and non-significant results for some malignancies. The number of factors that could be included in the analysis was limited by the data available in the insurance database; hence, clinical parameters such as HbA_1c_ and smoking could not be considered. All these factors could of course have possible confounding effects. Since body weight and body mass index (BMI) data are not available in the NHIF dataset, we cannot state how much weight loss or a difference in obesity contributed to the differences seen between the two classes of agents.

## Data Availability Statement

The raw data supporting the conclusions of this article will be made available by the authors, without undue reservation.

## Ethics Statement

The studies involving human participants were reviewed and approved by the Regional Ethical Board, University of Pécs, Clinical Centre (10338-5/2019/EKU) and National Institute of Health Insurance Fund (study license number: I043/122/2019). Written informed consent for participation was not required for this study in accordance with the national legislation and the institutional requirements.

## Author Contributions

GR was responsible for data acquisition, statistical analysis, and manuscript writing and preparation. ZK was responsible for data acquisition, statistical analysis, and preparation of figures and manuscript. GS was responsible for data interpretation and manuscript preparation. PK was responsible for funding, data interpretation, manuscript preparation, and proofreading. GJ was responsible for data interpretation, manuscript preparation, and proofreading. IF was responsible for data acquisition, statistical analysis, and manuscript preparation. ZS was responsible for funding and manuscript proofreading. GP was responsible for funding and manuscript proofreading. IW was responsible for funding, data interpretation, manuscript writing, preparation, and proofreading. GM was responsible for preparation of figures, manuscript writing, and proofreading. All authors contributed to the article and approved the submitted version.

## Funding

The study was funded by the Hungarian Diabetes Association and the Hungarian Society of Rheumatology.

## Conflict of Interest

GR and IF are employed by RxTarget Ltd., a company that is highly specialized in statistical analysis and data mining.

The remaining authors declare that the research was conducted in the absence of any commercial or financial relationships that could be construed as a potential conflict of interest.

## Publisher’s Note

All claims expressed in this article are solely those of the authors and do not necessarily represent those of their affiliated organizations, or those of the publisher, the editors and the reviewers. Any product that may be evaluated in this article, or claim that may be made by its manufacturer, is not guaranteed or endorsed by the publisher.
